# Supplementary material for: An abiotic source of Archean hydrogen peroxide and oxygen that pre-dates oxygenic photosynthesis
Source: Nat Commun. 2021 Nov 16;12:6611. doi: 10.1038/s41467-021-26916-2 (PMC8595356; doi:10.1038/s41467-021-26916-2)
Supplement: Supplementary file 1 — Supplementary Information [file 41467_2021_26916_MOESM1_ESM.pdf]

Supplementary Information for

An abiotic source of Archean hydrogen peroxide and oxygen that pre-dates oxygenic photosynthesis

This PDF file includes:

Supplementary Text

Supplementary Figures 1 to 10

Supplementary References

Supplementary Text

The calculation of the ratio of O_2 escaped to the headspace

O_2 concentrations in the headspace were attempted by using gas chromatography (GC), but no changes were apparent due to concentrations below the detection limit of the GC. Accordingly, we made an estimation of the amount of the O_2 escaped to the headspace based on the conversion process between O_2 and H_2O_2.

At the beginning of the second stage (5–120 min) in the kinetic release of ROS and O_2 at the abraded quartz-water interfaces under anoxic conditions, the maximum dissolved O_2 reached 0.15 mg L^{-1} in the 250-mL suspension, while the equivalent amount of dissolved O_2 was ~1.17 µmol. These values gradually decreased from T = 5 min to T = 120 min, with concentrations of 0.15 and 0 mg L^{-1}, respectively. There then was a rise of $[H_2O_2]$ from 4 to 8 µM (20 min < T < 120 min), which should be attributed to the conversion from the dissolved O_2 via combining with H• following the cycle reaction (i.e., $H\bullet + O_2 \rightarrow HO_2\bullet$ and $HO_2\bullet \rightarrow 0.5\ H_2O_2 + 0.5\ O_2$). The rise of $[H_2O_2]$ from 4 to 8 µM in the 250 mL suspension corresponds to 1 µmol of H_2O_2 produced (1 µmol = (8 – 4) µM × 0.25 L). This conversion consumed 85.33% of the dissolved O_2 in the suspension (=1/1.17). Thus, about 15% of the dissolved O_2 (1 - 85.33% = 14.67%) may escape to the headspace.

The flux of H_2O_2 in rivers and coastal zones

Based on the experimental H_2O_2 production (9.65 nmol·m^{-2}) at abraded quartz-water interface, we modelled H_2O_2 flux from a river and a sandy beach to an anoxic ocean

basin in the Archean, respectively.

Without surface vegetation coverage on the land, an Archean river should carry suspended sediments like today's Yellow River, which has a mean solid concentration of 25 kg m^{-3} (ref. [1]). In the suspended solids, quartz can account for up to 40 *wt.*% and its specific surface area is ~0.1 m^2 g^{-1} (ref. [2]), thereby the concentration of H_2O_2 in the river approaching the delta is 9.65 nM. Assuming the velocity of the river flow to the delta is 1 m s^{-1} (ref. [1]), the amount of H_2O_2 passed through the cross section (100 cm^2) per second is 96.5 nmol (Supplementary Figure 9a), i.e., 0.965 nmol cm^{-2} s^{-1}. The H_2O_2 flux to the delta is calculated to be 5.81×10^{14} molecules cm^{-2} s^{-1}.

The quartz on an Archean sandy beach would be under constant and intense friction by waves and tides. To estimate the in-situ flux of H_2O_2 of the coastal water at the Archean delta/shore, we use a small wedge-shaped unit of water column with a width of 1 cm and a depth of 0.2 m to calculate the H_2O_2 production in 1 year. If the angle of the slope near the sand beach is 7°, the volume of the wedge-shaped water column with a cross-section of 20 cm^2 is 1.63 L, which could carry about 407.5 g of quartz sand (Supplementary Figure 9b). The tumbling barrel experiments indicated that the increasing rate of the specific surface area of quartz in this specific hydrodynamic environment is 129.75 m^2 g^{-1} yr^{-1} (Supplementary Figure 8c). The total area of fresh surface produced in the water column in 1 year is 52863.52 m^2. By multiplying to the H_2O_2 production on the unit surface area of quartz, the amount of H_2O_2 generated in the water column is calculated to be 5.11×10^{-4} mol yr^{-1}. Thus, the in-situ flux of H_2O_2 diffusing across the section (20 cm^2) toward the open ocean is 4.87×10^{11} molecules

$cm^{-2}\ s^{-1}$.

The redox evolution of two locally oxidized environments in the Archean

The oxidants generated at the quartz-water interface would have led to the formation of oxygen oasis and driven the redox evolution in local aqueous environments in the Archean. To present a clear comparison with atmospheric photochemistry, we took the two calculated values of H_2O_2 flux into the quantitative model developed by McKay and Hartman (1991)[3] to simulate the redox evolution in Archean deltas and shores.

The dissolved ferrous iron was assumed to be the dominant reductant in Archean shallow seawater[4], and the concentration was set to 100 μM. As Fe(II) will be oxidized to Fe(III) rapidly by the H_2O_2 or its decayed product (O_2) when they diffuse across the oxic-anoxic interface, the reaction between Fe(II) and oxidants is not rate-limited but is determined by the diffusive transport of H_2O_2 through the oxic zone. Thus, we used the continuity equation (1) to describe the transport of H_2O_2 per unit area in the oxic zone,

$$\frac{d(L[H_2O_2])}{d_t} = F - \frac{L[H_2O_2]}{\tau} - K\frac{[H_2O_2]}{\frac{1}{2}L} \qquad (1)$$

where L is the horizontal length of the oxic front that is the path of the mechanical/chemical H_2O_2 transport (the L is replaced by Z, the vertical depth of the oxic zone that is caused by the photochemically produced H_2O_2 flux), $[H_2O_2]$ is the average concentration of peroxide, F is the seaward flux of H_2O_2 from abraded quartz-water interfacial reactions, τ is the lifetime of the peroxide in the oxic layer ($\tau = 0.1$ yr), and the term $K[H_2O_2]/(1/2L)$ approximates the rate of eddy diffusion of peroxide into

the ferrous edge ($K \approx 630$ cm^2 yr^{-1}), where it is rapidly consumed by reacting with Fe(II). As the water-soluble Fe(II) is titrated, the length of the pathway to the chemocline increases. The calculated results are shown in Supplementary Figure 10.

Here are steps of the numerical computation (personal communication with Christopher. McKay):

1. First set some initial conditions. Start with some initial tiny values, e.g.,

   $L = 2$ cm

   $[H_2O_2] = 1\times10^{-13}$ mol cm^{-3}

   Set an initial timestep,

   Dt = 0.001 yr

   It should be noted that these initial values would not determine the final results because these initial values will approach real values through iterations.

2. Then compute a time step in L, that is find a L_{new} from the L_{old}.

   $L_{new} = L_{old} + \mathrm{Dt} * \{\mathrm{K}\ [H_2O_2] / (0.5\ L_{old})\} / (1\times10^{-7})$

   The rate of H_2O_2 diffusing into the anoxic zone is K $[H_2O_2]$ / (0.5 L_{old}), and the amount of Fe(II) in 1 cm^3 of the seawater is 10^{-7} mol.

3. Then compute the new value of $[H_2O_2]$, treat $Y = L\ [H_2O_2]$ as the variable,

   $Y_{new} = Y_{old} + \mathrm{Dt} * \{Fa - Y_{old}/\tau - \mathrm{K}\ [H_2O_2] / (0.5\ L_{old})\}$

   Then $[H_2O_2] = Y/L$.

4. Then loop on steps 2 and 3.

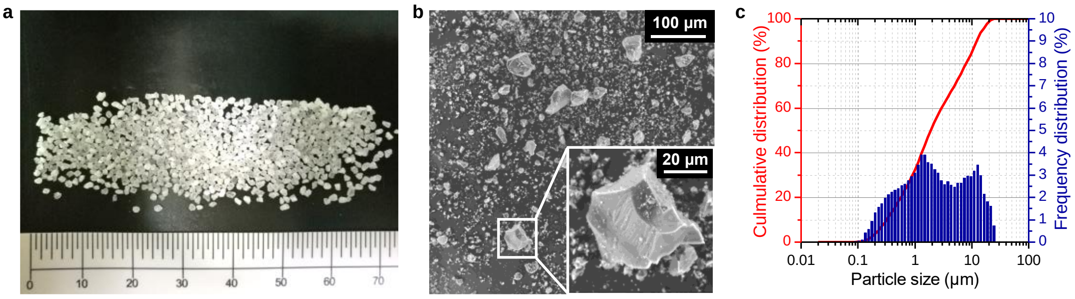

Supplementary Figure 1 The morphology and particle size of quartz before and after ball milling. a The digital photograph of raw quartz sands (0.25–0.6 mm). **b** The scanning electron micrograph of quartz ground in N_2 for 5 h. **c** The particle size distribution of the abraded quartz (with a median particle size of 0.002 mm).

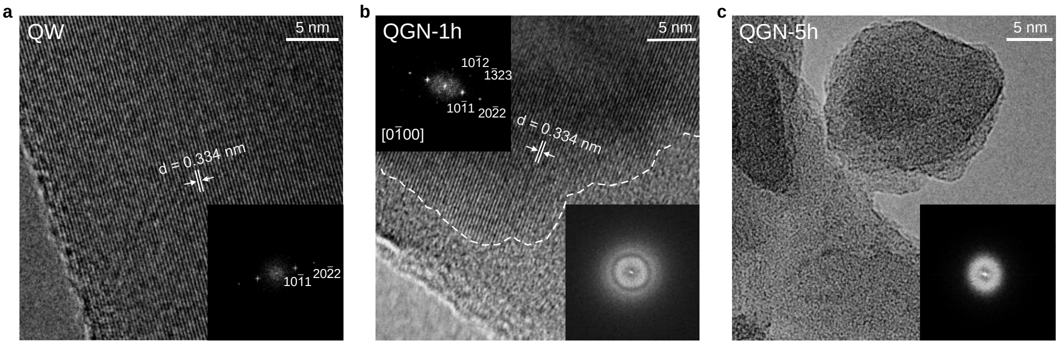

Supplementary Figure 2 Transmission electron micrographs of quartz before and after ball-milling in an ultrapure N_2 atmosphere for 1 and 5 h. a Quartz washed with 10 *wt.* % HF to remove surface impurities and to show surface without dangling bonds (QW). **b** Quartz ground by ball-milling in N_2 for 1 h (QGN-1h) shows an amorphous layer of > 5 nm thickness. **c** Quartz ground by ball-milling in N_2 for 5 h (QGN-5h) shows an enlarged amorphous layer due to the extended grinding time.

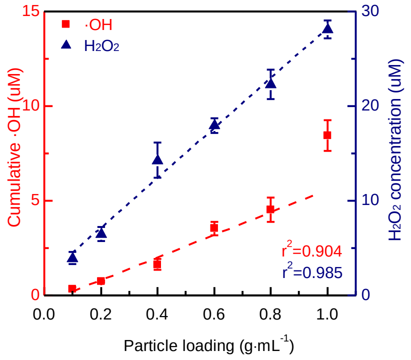

Supplementary Figure 3 The particle loading effect of quartz (ground in N_2 atmosphere for 5 h) on the production of •OH (red square) and H_2O_2 (blue triangle). The significant positive correlations (the r^2 values are 0.904 and 0.985 for [•OH] and [H_2O_2], respectively) between the quartz loading and the •OH and H_2O_2 concentrations suggests that the generation reaction of •OH and H_2O_2 is surface-controlled. The error bars present standard deviations of three independent replicates.

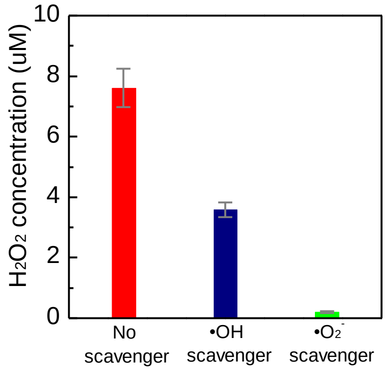

Supplementary Figure 4 The production of H_2O_2 at the presence of •OH and •O_2^- scavengers. The •OH and •O_2^-/HO_2• generated at the abraded quartz-water interfaces were scavenged by methanol and benzoquinone, respectively. At the presence of methanol (•OH scavenger), the H_2O_2 concentration was reduced from 7.61 μM to 3.59 μM, while almost no H_2O_2 generated at the presence of benzoquinone (•O_2^- scavenger). The results of the ROS scavenging experiments suggest that the H_2O_2 derived from the reactions between ≡SiOO• and water accounts for 47.13% of the total H_2O_2 production. The error bars present standard deviations of three independent replicates.

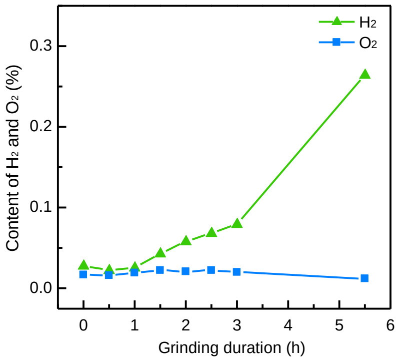

Supplementary Figure 5 Concentrations of H_2 (green triangle) and O_2 (blue square) in the headspace of the jar during grinding. After quartz sands were ground for 5.5 h, the content of H_2 in the headspace of the sealed ball-milling jar increased from a low background level to ~ 0.3%, while that of O_2 showed no obvious change.

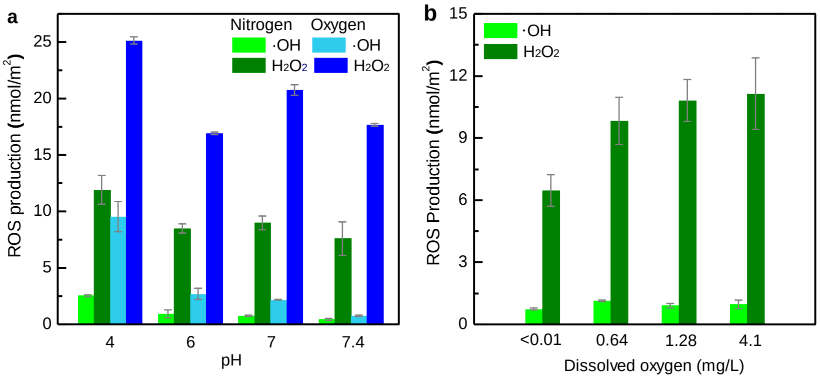

Supplementary Figure 6 Variation in production of •OH and H_2O_2 from abraded quartz under various aqueous and atmospheric conditions. a Productions of •OH and H_2O_2 at pH = 4.0, 6.0, 7.0, and 7.4 in the suspensions of quartz ground in N_2 (green bar) and O_2 (blue bar), respectively. Few •OH and H_2O_2 were detected in quartz washed with 10 *wt.* % HF. These experiments were conducted using waters with dissolved oxygen < 0.01 mg L^{-1}. **b** The effect of dissolved oxygen on the production of •OH and H_2O_2. The error bars present standard deviations of three independent replicates.

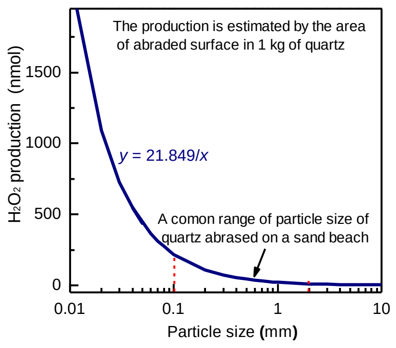

Supplementary Figure 7 The relationship between H_2O_2 production at the quartz-water interface and the particle size of quartz. For the fresh surface of 1 kg quartz with a grain size of 0.1 mm exposed in water, the H_2O_2 production is about 218 nmol.

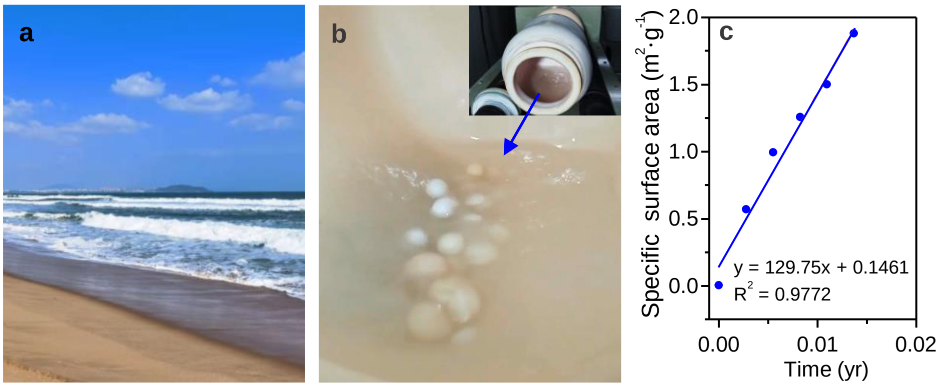

Supplementary Figure 8 The simulated physical erosion of quartz particles by waves and tides. a Waves on a sandy beach. **b** Photograph of the quartz in the tumbling barrel. **c** The increase in the specific surface area of quartz in the mild mechanical grinding conditions.

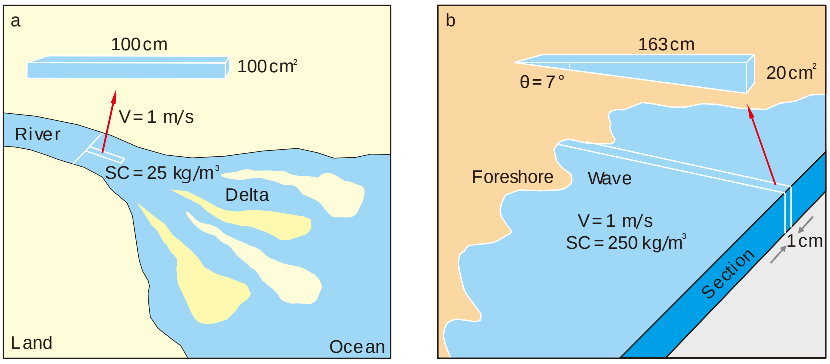

Supplementary Figure 9 The scheme of two models for the calculation of H_2O_2 flux. **a** The model for H_2O_2 flux from a river to the delta. At a flow velocity of 1 m s^{-1}, the volume of water column passed the cross section (100 cm^2) in 1 s is 10 L. The amount of H_2O_2 in this water column can be obtained via multiplying its volume by the H_2O_2 concentration (9.65 nM, see Supplementary Text). **b** The model for H_2O_2 flux at a sandy beach. Quartz is abraded intensely by high-energy waves and tides on the foreshore. The rate of in situ H_2O_2 generation in the water column is a function of the surface aera of the quartz abraded by currents (see Supplementary Text).

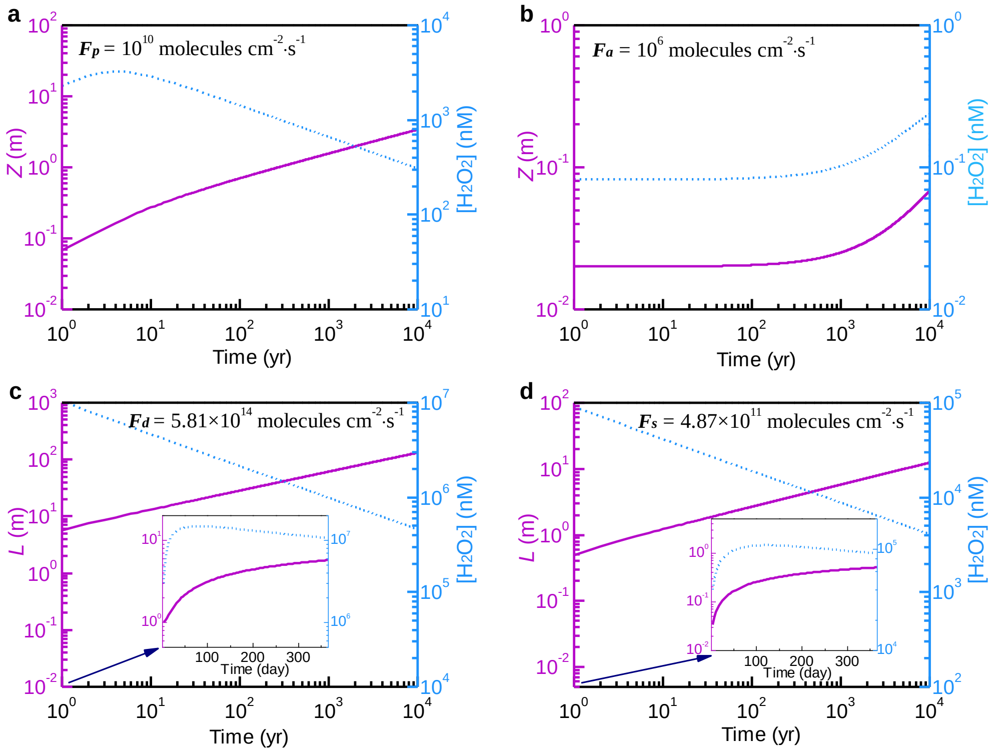

Supplementary Figure 10 The modelled time-course of H_2O_2 concentrations in local environments in the Archean. a-b A steady deposition of photochemically produced H_2O_2 into a previously anoxic basin when the H_2O_2 flux are 10^{10} molecules cm^{-2} s^{-1} (***F_p***, in relatively oxygenated, Proterozoic atmospheres, ref. [3]) and 10^6 molecules cm^{-2} s^{-1} (***F_a***, in the anoxic Archean atmospheres, ref. [5]), respectively. The results indicate that, in the Archean, merely with the deposition of photochemically produced H_2O_2, the [H_2O_2] in the surface water should be lower than 0.3 nM and the depth of this oxic zone is negligible ($Z < 0.1$ m), even after a 10^4 yr accumulation. This is consistent with the calculation of Haqq-Misra et al (2011)[5] which suggests that the resultant trace-level O_2 is insufficient to fuel early respiration (dissolved $O_2 > 3$ nM). **c-d** A steady transport of mechanical/chemical H_2O_2 into shallow seawaters in the Archean when the H_2O_2 flux is 5.81×10^{14} molecules cm^{-2} s^{-1} (***F_d***, in a river approaching the delta, see Supplementary Text) and 4.87×10^{11} molecules cm^{-2} s^{-1} (***F_s***, in seawater near shores, see Supplementary Text), respectively. After about 10^4 yr, the H_2O_2 concentration can be maintained at high levels (about 4×10^{-4} M) in the oxic zone near the delta (L = 100 m), and about 4×10^{-6} M for that near the shore (L = 10 m), respectively. Without considering the catalytic decomposition of H_2O_2 by peroxidases and catalases, the theorical H_2O_2 concentrations in both Archean locally oxidized environments are higher than the concentration of photochemically generated H_2O_2 in the upper layers of the present-day oceans (~10^{-7} M)[6,7]. Our calculations suggest that compared with the atmospheric photochemistry, the mechanical abrasion by currents on quartz could effectively deplete initial reductants and provide oxidants (H_2O_2 and O_2) for the phototrophs. These oxidants might have been enough to promote the development of oxygen tolerance and initiate the origin of oxygenic photosynthesis beneath an oxygen-poor atmosphere.

Supplementary References

1. Li, G., Wei, H., Yue, S., Cheng, Y. & Han, Y. Sedimentation in the Yellow River delta, part II: suspended sediment dispersal and deposition on the subaqueous delta. *Mar. Geol.* **149**, 113–131 (1998).
2. Tian, S. *et al.* Mineral composition and particle size distribution of river sediment and loess in the middle and lower Yellow River. *Int. J. of Sediment Res.* **36**, 392–400 (2021).
3. McKay, C. P. & Hartman, H. Hydrogen peroxide and the evolution of oxygenic photosynthesis. *Orig. Life Evol. Biosph.* **21**, 157–163 (1991).
4. Konhauser, K. O. *et al.* Iron formations: A global record of Neoarchaean to Palaeoproterozoic environmental history. *Earth Sci. Rev.* **172**, 140–177 (2017).
5. Haqq-Misra, J., Kasting, J. F. & Lee, S. Availability of O_2 and H_2O_2 on pre-photosynthetic Earth. *Astrobiology* **11**, 293–302 (2011).
6. Zika, R. G., Moffett, J. W., Petasne, R. G., Cooper, W. J. & Saltzman, E. S. Spatial and temporal variations of hydrogen peroxide in Gulf of Mexico waters. *Geochim. Cosmochim. Acta* **49**, 1173–1184 (1985).
7. Petasne, R. G. & Zika, R. G. Hydrogen peroxide lifetimes in south Florida coastal and offshore waters. *Mar. Chem.* **56**, 215–225 (1997).
