# Supplementary material for: An abiotic source of Archean hydrogen peroxide and oxygen that pre-dates oxygenic photosynthesis
Source: Nat Commun. 2021 Nov 16;12:6611. doi: 10.1038/s41467-021-26916-2 (PMC8595356; doi:10.1038/s41467-021-26916-2)
Supplement: Supplementary file 3 — Description of Additional Supplementary Files [file 41467_2021_26916_MOESM3_ESM.docx]

**Description for Additional** **Supplementary Files**

**Title:** Supplementary Video

**Description:** The simulated physical erosion of quartz particles by waves and tides.
